# Primary Hairy Cell Leukemia/Lymphoma of the Breast: A Case Report and Review of the Literature

**DOI:** 10.1155/2014/497027

**Published:** 2014-07-15

**Authors:** Monika Pilichowska, Ahmad Shariftabrizi, Ian Mukand-Cerro, Kenneth Miller

**Affiliations:** Department of Pathology and Laboratory Medicine and Department of Hematology-Oncology, Tufts Medical Center, Tufts University Medical School, Washington Street 800, Boston, MA 02111, USA

## Abstract

Hairy cell leukemia/lymphoma (HCL) is a rare B-cell neoplasm primarily involving spleen, bone marrow, and blood. However, other sites of primary involvement do occur and can present a diagnostic and therapeutic challenge. We present an unusual case of HCL involving predominantly the breast that was diagnosed as an incidental finding during an elective reduction mammoplasty in an otherwise healthy asymptomatic woman. Bone marrow performed for staging revealed limited involvement by HCL. Notably, there was no splenomegaly and/or involvement of other extramedullary sites. The peripheral blood revealed minimal involvement detected by flow cytometry. Extensive immunohistochemical studies supported by positive BRAF V600E mutational status confirmed the diagnosis of HCL. The patient remains asymptomatic without treatment one year following the diagnosis. This is the first case of a well-documented HCL presenting primarily in the breast in an asymptomatic patient. We review the literature on extramedullary, extrasplenic involvement by HCL and discuss the diagnostic challenges as well as the utility of immunohistochemistry and molecular studies in the diagnosis of atypical presentations of HCL.

## 1. Introduction

Hairy cell leukemia (HCL) was first identified as a distinct clinical and histopathologic entity by Bouroncle et al. in 1958. He described an indolent disorder and characterized its clinical course, pathologic features, treatment options, and prognosis [1, 2]. Despite the fact that splenectomy was already proven to be beneficial, the true nature of the neoplastic cells was unknown until the development of the newer immunophenotypic methodologies in the mid-1970s [3]. HCL is now recognized as a neoplasm of mature B-cell involving blood, bone marrow, and splenic red pulp. HCL affects adults with median age of 50 years and shows a male to female predominance of 4 : 1; it usually has an indolent, chronic course characterized by progressive pancytopenia, splenomegaly, and frequently monocytopenia. Circulating small monocytoid B-cells “hairy cells” with the characteristic hair like cytoplasmic projections are generally rare and can be difficult to identify on the peripheral blood smear [4].

Although rarely HCL can affect other organs including the mediastinum, retroperitoneal and paravertebral nodes, skin, gastrointestinal tract, nervous system, and ocular cavity [5, 6], we here present a case of HCL involving the breast, which was diagnosed as an incidental finding at the time of an elective reduction mammoplasty in an otherwise asymptomatic woman.

## 2. Case History

A 44-year-old woman who was in her usual state of health underwent elective bilateral breast reduction surgery. Morphologic examination of the random tissue samples performed as a part of routine pathologic examination revealed an atypical lymphoid infiltrate in the left breast consistent with a low grade B-cell lymphoma. The right breast was not affected. The patient was referred to our hospital for further evaluation and treatment.

At the time of her initial visit, she was asymptomatic. She reported a good appetite and her weight was stable. She did not report any breast fullness, pain, or nipple discharge. She was performing regular self-breast exams, which were normal, and had two prior normal mammograms a year and six months prior to the elective surgery. Her personal and family history was noncontributory. Notably, there was no history of familial cancer. She did not smoke, use alcohol, drugs, and/or medications. She had a diagnosis of Factor XI deficiency made as a part of the preoperative workup. On the physical exam, she had no adenopathy, splenomegaly mucosal, or skin lesions and the remainder of her past medical history and her physical was noncontributory. Her peripheral blood counts were normal without monocytopenia or other cytopenias with WBC (×10^3^/mm^3^) 6.6, RBC (×10^6^/mm^3^) 4.62, and Plt (×10^3^/mm^3^) 257 and normal differential count. All other laboratory values were within normal limits.

## 3. Material and Methods

The diagnostic slides from the material obtained from the left breast surgery were reviewed in consultation. These slides included H&E slides and immunohistochemical stains used for the evaluation of atypical lymphoid infiltrate. Tissue block corresponding to the slide containing most of the lymphoid infiltrate was obtained and additional immunohistochemical stains were performed at Tufts Medical Center for confirmation of the diagnosis. The immunohistochemical stains were performed on the VENTANA automated system according to the established protocols. All the antibodies were used at standard dilution and included (abbreviated list) CD20, PAX5, TRAP, CD25, bcl-1 (Cyclin D1), DBA.44 (CD72), Annexin-A1, CD3, CD5, Keratins, S-100, CD163, CD43, bcl-6, bcl-2, and CD10.

To rule out mantle cell lymphoma fluorescent in situ hybridization (FISH) for t(11; 14) was performed on formalin fixed, paraffin embedded tissue sections. Commercially available double fusion probe (Vysis, Abott Molecular) was used for this purpose. Subsequently the tissue was subjected to BRAF V600 mutational analysis for genotypes c.1798G and c.1799T. The analysis was performed on genomic DNA extracted from formalin-fixed, paraffin-embedded tissue block utilizing mutation detection technology by single base extension sequencing (SNaPshot, Applied Biosystems). Extension products were visualized using the ABI 3730 DNA analyzer.

At the time of clinical visit, peripheral blood was obtained for morphology and flow-cytometric immunophenotyping and bone marrow aspirate and biopsy was performed. Flow-cytometric immunophenotyping was performed on the Beckman Coulter BC 500 flow cytometer and utilizing BC supplied antibodies for CD3, CD5, CD4, CD7, CD8, CD19, CD20, CD10, CD11c, CD103, kappa, lambda, CD45, CD13, CD33, CD34, CD117, CD14, and HLA-DR. The bone marrow biopsy was fixed in acetic acid-zinc formalin (AZF), decalcified, and processed for light microscopy according to standard protocols. Bone marrow aspirate was dry tap. In addition to standard examination limited immunohistochemical panel including CD138, bcl-6, Pax5, and CD20 was performed on the bone marrow biopsy to evaluate for involvement by a B-cell lymphoproliferative disorder.

## 4. Results 

**Figure 1:**
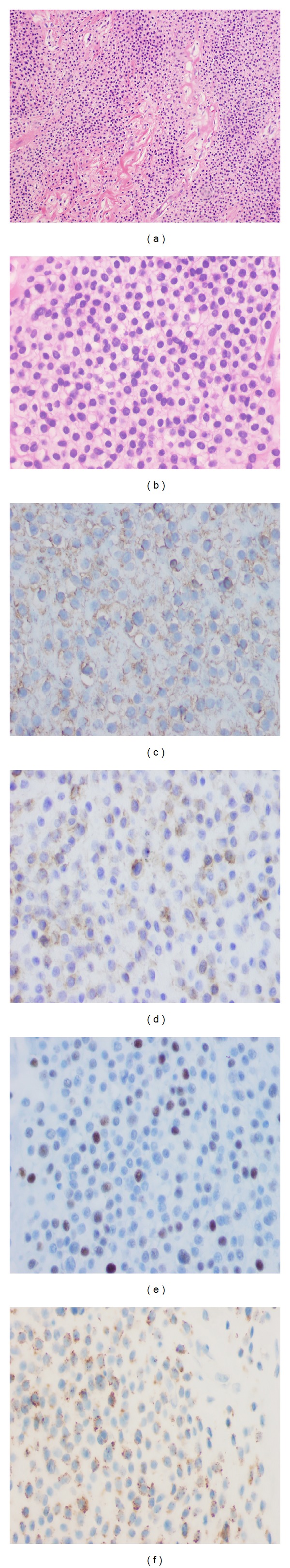
(a) Morphologic examination reveals interstitial lymphocytic infiltrate of small mature lymphocytes (H&E ×200). (b) The neoplastic cells have round nuclear contours, condensed chromatin, inconspicuous nucleoli, and ample pale cytoplasm (H&E ×400). Immunohistochemical staining reveals that the neoplastic cells are CD20 positive B-cells (c) showing cytoplasmic staining for Annexin (d), nuclear staining for Cyclin D1 (e), and cytoplasmic TRAP (f). (Immunoperoxidase ×400).

On microscopic examination, the breast tissue revealed a relatively monotonous, diffuse, and focally more prominent interstitial infiltrate of small mature appearing lymphocytes. The lymphocytes had round nuclear contours, condensed chromatin, and indistinct nucleoli. Ample pale cytoplasm was noted. There were no admixed large cells. Associated breast tissue showed focal areas of fibrosis and benign fibrocystic changes. There were no periductal lymphoid aggregates and the lymphoid infiltrate was interductal and not associated with epithelial component (Figures 1(a)-1(b)). Immunohistochemical staining revealed that the infiltrating lymphocytes were CD20 and PAX5 positive B-cells showing variable nuclear expression of Cyclin D1 as well as cytoplasmic Annexin-A1 and TRAP (Figures 1(d)–1(f)) and weak CD25. DBA.44 staining was inconclusive. All other immunohistochemical stains performed were negative. Fluorescent in situ hybridization (FISH) for t(11; 14) to rule out mantle cell lymphoma was negative. The overall morphologic and immunohistochemical features were consistent with HCL.

**Figure 2:**
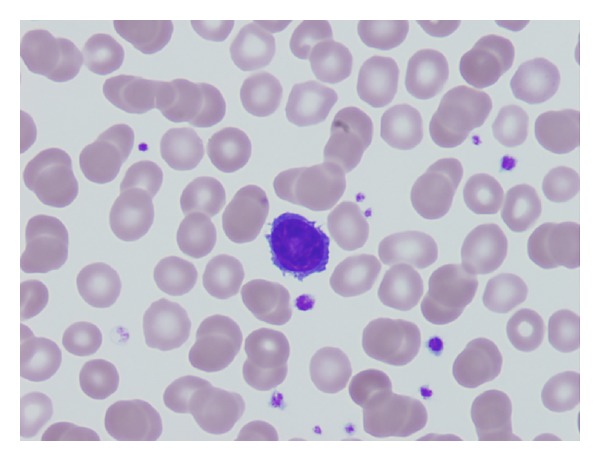
Peripheral blood smear with occasional small lymphocytes showing ample pale cytoplasm and cytoplasmic projections “hairy cells.”

Evaluation of the peripheral blood smear revealed rare, less than 1%, small to medium sized lymphocytes with eccentric nuclei, round nuclear contours, mature chromatin without nucleoli, and ample amount of cytoplasm showing cytoplasmic projections. These cells were cytomorphologically consistent with “hairy cells” (Figure 2). Flow cytometry analysis performed on the peripheral blood revealed infrequent B-cells (<1% of total). Clonality of these B-cells could not be established.

**Figure 3:**
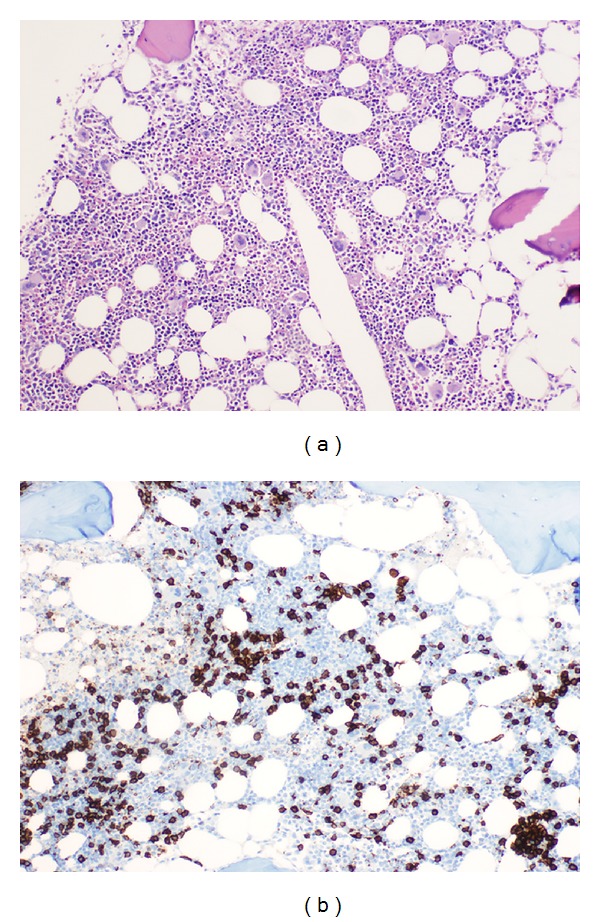
(a) Bone marrow biopsy shows a rather inconspicuous diffuse infiltrate of small mature lymphocytes (H&E ×200). (b) Immunohistochemical staining for CD20 reveals B-cell nature of the infiltrate. (Immunoperoxidase ×200).

Evaluation of the bone marrow biopsy revealed normocellular for age marrow. There was maturing trilineage hematopoiesis with normal myeloid to erythroid ratio of 3 : 1. There was a subtle, diffuse interstitial infiltrate of small mature lymphocytes constituting overall up to 10% of total bone marrow cellularity (Figure 3(a)). Immunohistochemical staining revealed that the infiltrating lymphocytes were PAX5 and CD20 positive B-cells (Figure 3(b)). The overall morphologic and immunophenotypic features were consistent with marrow involvement by hairy cell leukemia. In addition, reticulin stain revealed moderate, diffuse reticulin fibrosis, a finding known to be associated with HCL. Flow cytometric examination performed on the bone marrow aspirate smear revealed a minute population of B-cells (<1%) with marked predominance of kappa versus lambda surface light chains (kappa : lambda > 10 : 1) highly suggestive of involvement by a clonal B-cell process with kappa light chain restriction. BRAF V600 mutational analysis (performed on the breast tissue material) for genotypes c.1798G and c.1799T was positive for BRAF V600 mutation.

## 5. Discussion

The World Health Organization classifies HCL as mature B-cell neoplasm with predilection for splenic involvement and certain immunophenotypic characteristics [7]. Classically, diagnosis of HCL was confirmed by tartrate-resistant acid phosphatase activity, although the standard practice today is immunophenotyping by flow cytometry where HCL is characterized by the expression of B-cell antigens CD19, CD20, and CD22 in addition to coexpression of the surface antigens CD11c, CD25, and CD103. Hairy cells generally lack CD5, CD10, CD21, and CD23; also immunohistochemical stains for DBA44, Cyclin D1, and Annexin can be performed on tissue sections and are usually positive. The immunohistochemistry is indispensable when no fresh tissue is available for flow cytometric immunophenotyping. Cytologic features of the HCL include mature lymphocyte with small size and round nuclear contour with a condensed chromatin and indistinct nucleolus, abundant pale cytoplasm, and circumferential cytoplasmic projections. The finding of hairy cells having nuclei widely separated by abundant cytoplasm, resulting in the so-called “fried-egg” appearance, can be sometimes a clue to the diagnosis along with the splenomegaly; the bone marrow is involved in nearly all patients with HCL and is hypercellular in most cases [4, 7]. Typical sites that are involved by HCL are the bone marrow and splenic red pulp; the disease can also be more widespread and involve extramedullary sites such as the central nervous system, gastrointestinal and urogenital tracts, heart, lungs, skeletal muscle, skin, thymus, and thyroid [5, 6, 8]. Involvement of the bone marrow in HCL with associated reticulin fibrosis results characteristically in hypocellular aspirate smears or dry tap. Approximately half of patients present with pancytopenia, while the remaining cases might have varying combinations of anemia, neutropenia, and thrombocytopenia [8]. Our patient had rare circulating hairy cells and limited bone marrow involvement but no cytopenia or splenomegaly.

HCL involving the breast is exceedingly rare and only one prior case report has been published to date by Farkash et al. which was a case of breast HCL concurrent with ductal carcinoma in situ (DCIS) [9]. This published case represented an advanced HCL disease given the fact that HCL involved the lymph nodes and extensively bone marrow with patient reporting bone pain. Therefore, the breast tissue involvement occurred most likely in process of systemic spread of the disease since it is known that lymph node, skeletal, and other tissue involvement can occur at an advanced stage of disease. Our case did not show any evidence of systemic disease except limited bone marrow involvement. Notably there was no splenomegaly and the physical examination was normal. On morphologic examination of the breast tissue the lymphoid infiltrate had a diffuse but indolent appearance and was situated in the stroma with no ductal or periductal lesions. It was composed of small monotonous lymphocytes with prominent pale cytoplasm. The latter provided a clue for diagnosis and immunohistochemical workup. Immunophenotypically the neoplastic cells were CD20 and PAX5 positive B-cells characteristically exhibiting nuclear staining for Cyclin D1 and cytoplasmic positivity for Annexin, TRAP, and weak CD25. BRAF V600 mutation has been recently described for positive identification of HCL, considering very high specificity and sensitivity of this test for HCL [10, 11]. We believe that the finding of BRAF V600 mutation was helpful to confirm the diagnosis of HCL in our case.

The most common nonepithelial neoplasms involving the breast are lymphoid malignancies, with primary breast lymphomas (PBL) representing 0.5% of malignancies of the breast. Most of the PBL are B-cell lymphomas and T-cell lymphomas which rarely involve the breast. The two most common lymphomas involving the breast are diffuse large B-cell lymphoma followed by extranodal marginal zone lymphoma of mucosa-associated lymphoid tissue (MALT lymphoma). B-lymphoblastic lymphoma, Burkitt's lymphoma, peripheral T-cell lymphoma, rarely, classic Hodgkin's lymphoma, and follicular lymphoma are other PBLs that are less frequently observed. Secondary breast lymphomas (SBL) are defined as the lymphomas with the breast being a minor dissemination site and are most commonly follicular lymphoma. The differential diagnosis for HCL regardless of the site of involvement includes all forms of mature B-cell lymphomas including marginal zone lymphoma (MZL), splenic lymphoma with villous lymphocytes (SLVL), B-cell prolymphocytic leukemia (B-PLL), and very rarely mantle cell lymphoma (MCL) or atypical CD5 negative chronic lymphocytic leukemia/lymphoma (CLL/SLL) and lymphoplasmacytic lymphoma. Follicular center cell lymphoma is also a possibility which can be ruled out based on the bcl-6 and CD10 positivity [12–14].

In summary, we present a first case of HCL presenting in the breast as incidental finding. The diagnosis was based on cytomorphology, immunophenotype, and presence of BRAF V600 mutation.
